# A Method of Protein Model Classification and Retrieval Using Bag-of-Visual-Features

**DOI:** 10.1155/2014/269394

**Published:** 2014-09-01

**Authors:** Jinlin Ma, Ziping Ma, Baosheng Kang, Ke Lu

**Affiliations:** ^1^School of Information and Technology, Northwest University, Xi'an 710120, China; ^2^School of Mathematics and Information Science, North University of Nationalities, Yinchuan 750021, China; ^3^College of Computing & Communication Engineering, University of Chinese Academy of Sciences, Beijing 100049, China

## Abstract

In this paper we propose a novel visual method for protein model classification and retrieval. Different from the conventional methods, the key idea of the proposed method is to extract image features of proteins and measure the visual similarity between proteins. Firstly, the multiview images are captured by vertices and planes of a given octahedron surrounding the protein. Secondly, the local features are extracted from each image of the different views by the SURF algorithm and are vector quantized into visual words using a visual codebook. Finally, KLD is employed to calculate the similarity distance between two feature vectors. Experimental results show that the proposed method has encouraging performances for protein retrieval and categorization as shown in the comparison with other methods.

## 1. Introduction

The classification and retrieval of protein models is widely applicable in biomedical science. Biologists have a great demand for protein retrieval and classification tools to identify the functions of unknown proteins and to discover new functions of known proteins.

The most widely used protein structure classification systems are CATH [1] and SCOP [2], both of which are created by experts based on their experiences. With the rapid growth of the 3D protein structures, the artificial classification has been unable to meet the demand. It is desirable to do classification and retrieval in a more automated way. So, more and more researchers are dedicated to studying automatic classification methods which are based on the biological function of the protein molecules.

The protein molecules are of some specific shape which can be described by their biological function, for example, the amino acid sequences and 3D structures. According to the different biological functions, there are three kinds of methods for protein retrieval and classification. They are respectively based on molecular sequence, protein secondary structure (SS) elements, and 3D structural coordinates.

The methods based on the molecular sequence aim to determine the amino acid sequences, since the amino acid sequences of proteins are easily understandable and simple to classify. The methods include FASTA [3], BLAST [4], PSI-BLAST [5], and Hidden Markov Models [6].

In most cases, the protein is represented by a set of SS elements. So many researchers are devoted to designing different algorithms to represent vector features by SS elements or to obtain the similar distance between the SS elements. Milledge et al. [7] created a geometrical hashing using interatomic distance to identify the triples of atoms. Zotenko et al. [8] mapped the structure to a high-dimensional vector and utilized distance between the corresponding vectors to approximate the structural similarity. Feature vectors are extracted from contact regions of the secondary structure elements (SSEs) by Aung and Tan [9]. Camoglu et al. [10] used R-Tree in indexing the vector features which are represented by SS. Cantoni et al. [11] proposed a protein structural motif retrieval approach based on Generalized Hough Transform, which evaluates the triplet of the Secondary Structure by midpoints distance. In literature [12], Mavridis et al. compared the performance of six algorithms including Contact Maps, 3DZernike, Group Integration, Genocrypt, Spherical Trace Transform, and 3DBlast by classifying protein structures according to the CATH superfamily classification. The experimental results showed that contact maps and 3DBlast are conceived specifically to compare the structures of proteins.

The methods based on the 3D structure coordinates try to describe proteins shape by identifying or comparing structural alignment. MAMMOTH [13] modeled portion of the target structure and compared protein structure with an arbitrary low-resolution protein model. TM-align [14] identified the best structural alignment by protein pairs and Dynamic Programming. FAST [15] compared the intramolecular residue-residue relationships of two structures by using a directionality-based scoring scheme. In order to reduce the coordinate-independent space of protein structures, Holm and Sander [16] proposed the optimal pairwise structural alignment algorithm using Monte Carlo. Shindyalov and Bourne [17] studied heuristics combinatorial extension and similarity evaluation of structural alignment path algorithm.

In recent years, there appeared a method based on image distance matrices. Ankerst et al. [18] introduced 3D shape histograms algorithm to compare protein models or molecules. Chi et al. [19] compared protein structures by using signatures extracted from image-based distance matrices and multidimensional index. Yeh et al. [20] compared the protein models from multiple 3D projection views. The image-based retrieval methods exhibited a higher degree of precision than the three kinds of traditional methods.

In this paper, we propose an image-based protein retrieval and classification method using SURF algorithm to extract features and *k*-means to cluster the features, thus generating a codebook. We use histogram determined by BOVF (bag-of-visual-features) vectors to represent the characteristics of the identified models.

We construct an image-based method to avoid exhaustive search for the molecular sequence, structure coordinates, and chain structure alignments. Our major contribution is to propose an efficient protein models retrieval and classification method by using bag-of-visual-feature. The performance is exciting. Our experimental retrieval precision is 96% on average.

This paper is organized as follows.  Section 2 discusses the related algorithm for SURF and bag-of-feature and then details the proposed method. Experimental results are represented and analyzed in Section 3. In the final section we conclude this paper.

## 2. Materials and Methods

### 2.1. Bag-of-Features

The bag-of-words method was first used in document retrieval and applied in 3D shape retrieval and categorization, due to its many advantages such as simplicity, flexibility, and efficiency. The bag-of-features was first proposed by Liu et al. [21] for both global comparison and partial matching. It relies on the extraction of spin image signatures which are later grouped in clusters. Yu et al. [22] built an effective image retrieval system based on the bag-of-features model. They, respectively, integrated the SIFT and LBP descriptors and the HOG and LBP descriptors and proposed the patch-based integration and image-based integration models. The experimental results showed that the image-based SIFT-LBP integration clustering by weighted *k*-means algorithm achieves the best performance. A simple, novel, yet powerful approach was presented for background subtraction by bag-of-features [23]. They supposed that encoding the local color and texture information can effectively attenuate the texture variations in the background scenes and then domain-range features were encoded in the soft-assignment coding procedure which is decided by the appropriate kernel variances. Nanni and Lumini [24] applied bag-of-features and heterogeneous set of texture descriptors for object recognition. The proposed method is based on a simple exhaustive extraction of subwindows and classification of random subspace by support vector machine (SVM) and can reduce dimensions by the principal component analysis. Moreover, Zhou et al. [25] proposed a method for scene classification using a multiresolution bag-of-features model. The bag-of-features approach can be also applied in music classification [26], distinction text between handwritten and machine-printed [27], and noise filter [28] and so forth.

The bag-of-features has also been applied to local feature 3D shape retrieval and classification. Ohbuchi et al. [29] extracted local features from each range image of different views using the Scale Invariant Feature Transform (SIFT) algorithm [30] for retrieving rigid models and articulated models. In [31], a novel framework is employed to combine spectral clustering with region growing based on fast marching 3D object categorization.

BOF was also used in analyzing medical images and computer-aided diagnosis (CAD). Shen et al. [32] proposed a human epithelial type 2 (HEp-2) classification framework using intensity order pooling based on gradient feature and bag-of-words. The pooled gradient feature extracted by the intensity orders of local grid points is rotationally invariant, which outperformed SIFT feature significantly. Wang et al. [33] investigated two issues of bag-of-feature strategy for tissue classification and developed a novel algorithm named Joint-ViVo to learn the vocabulary and visual word weights jointly. The test results showed that the algorithm is better than the state-of-art methods on classifying breast tissue density in mammograms and lung tissue in high-resolution computed tomography (HRCT) images and identifying brain tissue type in magnetic resonance imaging (MRI).

### 2.2. Speed-Up Robust Features (SURF)

SURF was proposed by Bay et al. [34]. It is based on sums of 2D Haar wavelet responses and Hessian matrix based interest point's detector and it makes an efficient use of integral images, which are a robust local feature detector and descriptor that can be used in computer vision tasks like object recognition for 3D reconstruction. Though SURF is partly inspired by the SIFT descriptor, it is several times faster and more robust than SIFT.

Gui et al. [35] proposed a novel point-pattern matching method based on the SURF and Shape Context. They applied the SURF bidirectional matching to match the feature points in two images preliminarily and then calculated Shape Context descriptors of the feature points. Experimental results show that the method can eliminate the incorrect matching point pairs and improve the accuracy of point-pattern matching. A fully affine invariant SURF algorithm was proposed by Pang et al. [36], which has the affine invariant advantage of ASIFT and the efficient merit of SURF. Alcantarilla et al. [37] proposed a descriptor named Gauge-SURF which is evaluated relative to the gradient direction at every pixel. Because of the use of integral images, the descriptors are fast and robust.

Recently, SURF was used in iris retrieval and recognition. A hierarchical approach was proposed to retrieve an iris image from a large iris database [38]. The approach is a combination of both iris color and texture and the iris texture features are obtained by SURF algorithm. Mehrotra et al. [39] proposed a robust segmentation and an adaptive SURF descriptor for iris recognition. In their method, the adaptive strip transformed from the annular region between the iris and pupil boundaries is enhanced using a gamma correction approach. Then, features are extracted from the adaptive strip using SURF. Feulner et al. [40] presented a method for automatically estimating the body region of a CT volume image. The method is based on 1D registration of histograms of visual words, which serves as a description of a CT slice. The SURF descriptor was extended to *N* dimensions named *N*-SURF. Because of its simpler and efficient functioning, they used 2D upright SURF descriptors for estimating the body region.

### 2.3. The Proposed Method

**Figure 1:**
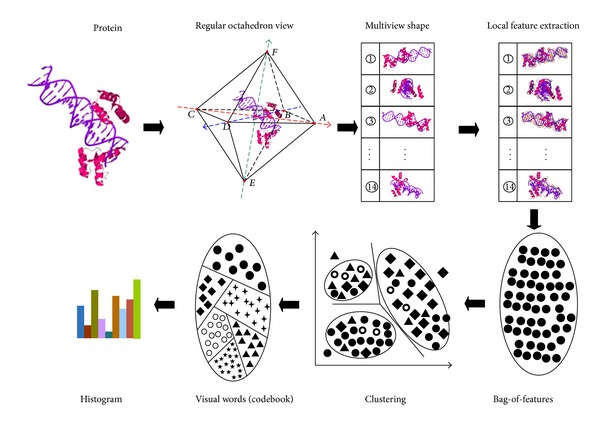
An illustration of our method (PDB code “1hdd”).

**Figure 2:**
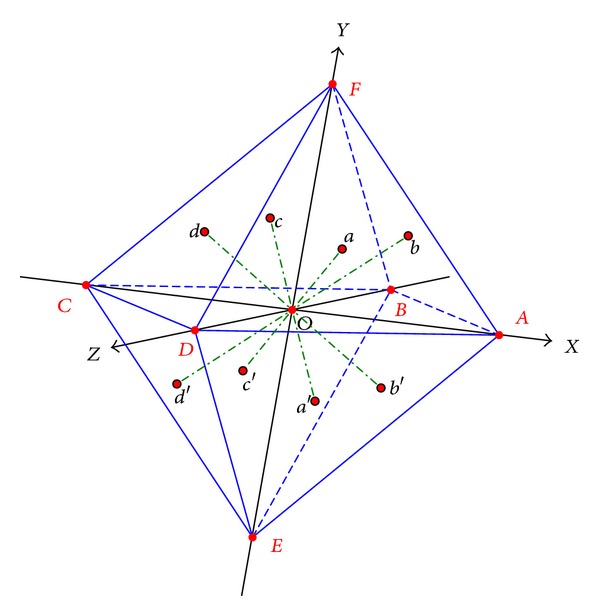
The multiviews are captured from the vertices and the planes on a given regular octahedron.

The key idea of our method is to extract the features of proteins and measure the visual similarity between proteins. Our algorithm is implemented subsequently in four steps, as shown in Figure 1.

(*1) Multiview Rendering*. Render multiview images of the protein from different perspectives. The viewing angle is determined using the vertices and planes of a given octahedron structure surrounding the protein, as shown in Figure 2. 

(*2) Local Feature Extraction*. Extract the local visual features of the multiview images by using SURF algorithm. Then, for each view, we calculate the SURF descriptors. 

(*3) Visual Words Generation and Word Histogram Construction*. Generate visual words from feature vectors using a visual dictionary (i.e., the codebook). A visual dictionary can be got by *k*-means clustering and so each local feature shall be represented as a discrete form. The frequencies of visual words are counted and stored into a histogram, which becomes the feature vector of the corresponding protein model. 

(*4) Distance Computation*. The dissimilarity among a pair of feature vectors (the histogram) is computed by the Kullback-Leibler divergence (KLD).

#### 2.3.1. Multiview Rendering

The multiview shapes are captured from the six vertices and the eight planes on a given regular octahedron. Figure 2 shows the six vertices and the eight planes on the octahedron. Along the *x*+, *x*−, *y*+, *y*−, *z*+, and *z*− axes, we capture the protein's right view, left view, top view, bottom view, front view, and rear view. Along the normal direction of each plane, we get the protein's eight oblique views. The size of the captured image is set as 100 × 100 pixels.

**Figure 3:**
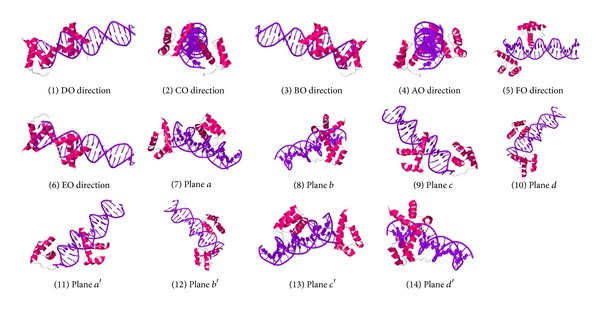
Fourteen views of protein “1hdd” (PDB code).

As we mentioned above, 14 different views still exist for a protein.  Figure 3 illustrates all the 14 views of protein structure encoded as “1hdd” with PDB code after adjusting the viewing perspective.

To reduce the interference in operating and standardizing the rendering process, we write a program for view capturing from CATH and SCOP in the PDB. The program can automatically load protein models, rotate models, capture images, and save images following a certain naming rule. Of course, the protein models are also selected automatically by the program in advance.

#### 2.3.2. Local Feature Extraction

After the range images are rendered, the SURF algorithm is applied to each of the range images to detect interest points and then to extract SURF descriptors, as presented in [30]. The SURF algorithm detects interest points and then computes features at these interest points. The SURF firstly finds positions of features that are salient. The saliency detection is based on a multiscale and multiorientation Fast-Hessian detector and distribution-based descriptor for gray-level change so that each SURF feature can encode this information. The SURF descriptor is calculated using the OpenSURF C++ source code by Evans [41].

**Figure 4:**
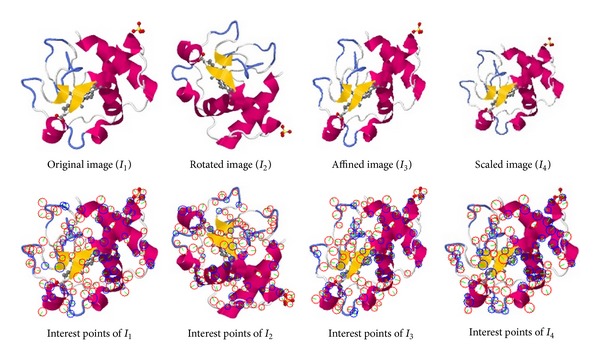
Interest points of the SURF algorithm are robust.

Figure 4 shows examples of an interest point generated and its images that are rotated, affined, and scaled. The numbers of interest points of SURF algorithm are 113, 112, 102, and 93, as shown in the second row. The interest points appear at similar locations in these four images in spite of the geometrical transformations. This robustness against geometric transformations contributes to the protein model retrieval performance.

**Figure 5:**
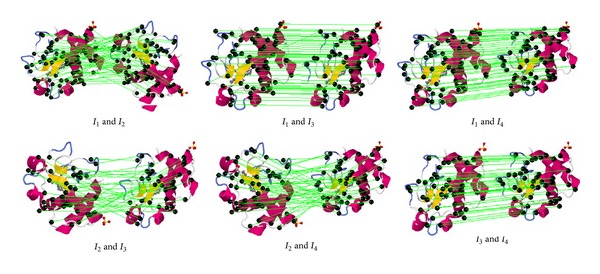
Interest points matching.

Figure 5 shows the examples of SURF interest points match of images in Figure 4. The numbers of interest points matching are 59, 52, 54, 35, 45, and 40. Every image is successfully matched to a certain feature point. Because of the different interest feature points (in number, size, and position) extracted by SURF, the numbers of the feature points that match are different.

#### 2.3.3. Visual Words Generation and Word Histogram Construction

It is time consuming to compare model's local SURF feature directly, especially for the large number of views. Therefore it is necessary to quantize the SURF descriptors extracted from a multiview image into visual words. Firstly, a visual codebook is generated by using off-line *k*-means clustering of the features of every view. Then, the codebook is searched linearly to find a visual word closest for the feature. As a result, the feature vectors of visual words are selected through the centers of the clusters (called barycenter), and the number of the clusters determines the codebook size.

After generating the codebook, we should construct a word histogram over the codebook, which is also an off-line process. The word histogram is constructed by counting the frequencies of visual words. Each view is represented as a word histogram which is the features extracted by bag-of-visual-features. So the protein model's histogram is produced by combining every view image's histogram of the protein model.

#### 2.3.4. Distance Computation and Range Models Matching

The last step of our method is the distance computation (also called models matching) between two models. A distance among a pair of feature vectors (the histogram) is computed by using the Kullback-Leibler divergence (KLD). The KLD is not a distance metric, for it is not symmetric. Consider

(1) $$\begin{array}{c} D\left(x,y\right)=\overset{n}{\underset{i=1}{\sum }}\left(y_{i}-x_{i}\right)\ln\frac{y_{i}}{x_{i}}, \end{array}$$

where, **x** = (*x*_*i*_) and **y** = (*y*_*i*_) are the feature vectors and *n* is the dimension of the vectors.

## 3. Results and Discussion

In order to evaluate the efficacy and generalization capacity of the proposed method, we tested it with several different retrieval and categorization tasks. The first experiment compares the performance of our method and the compared method in terms of their capability for classification. The second experiment tests the impact of the numbers of the clusters on the methods' retrieval capability. The last experiment tests the influence of the size of the training data on the method.

We implement the experiments in Matlab R2010a, while the SURF and the *k*-means code is written in C++. All algorithms are run under windows 7 32 bit on a personal computer with a Core 2 Quad 2.66 GHz CPU, 3.00 GB DDR2 memory, and a 512 MB ATI Radeon HD4600 graphics card.

To evaluate the method's efficiency, we measured the feature extracting time. We experimented on the computation time in a 2,000 protein models database which has images of 28,000 views. The computation time for SURF feature extracted algorithm is about 219.48 seconds (an off-line process). For clustering the features by *k*-means and generating the histograms by bag-of-visual-features, it takes 42.49 seconds and 0.66 seconds by an off-line process for the same 2,000 protein models database.

### 3.1. Classification

In the first experiment, we evaluate the classification performance of our approach by using protein models database of SHREC 2010 [12], which includes 1000 protein structures chosen from 100 CATH 3.3 superfamilies. In the dataset each superfamily consists of at least 10 structures, and each structure contains at least 50 amino acids. We use two different ways (nearest neighbor, ROC plot) to test the performance of our method by comparing with 3DBlast, 3DZernike, GENOCRIPT, Contact Maps, Group Integration, and Spherical Trace Transform methods.

Nearest neighbor was counted as a correct prediction when the first protein of each ranked list was found to be a member of the same superfamily as the query.

ROC plot (receiver operating characteristic) is a graphical plot which illustrates the true positives rate against the false positives rate. The area under the curve (AUC) is a single numerical performance measure of each ROC plot. The perfect value of AUC is 1.0 [12].

Table 1 summarizes the retrieval rate for all the methods. The value of the comparison algorithm is from literature [12]. According to Table 1, the correct prediction of the proposed method is better than the comparison algorithm.

**Figure 6:**
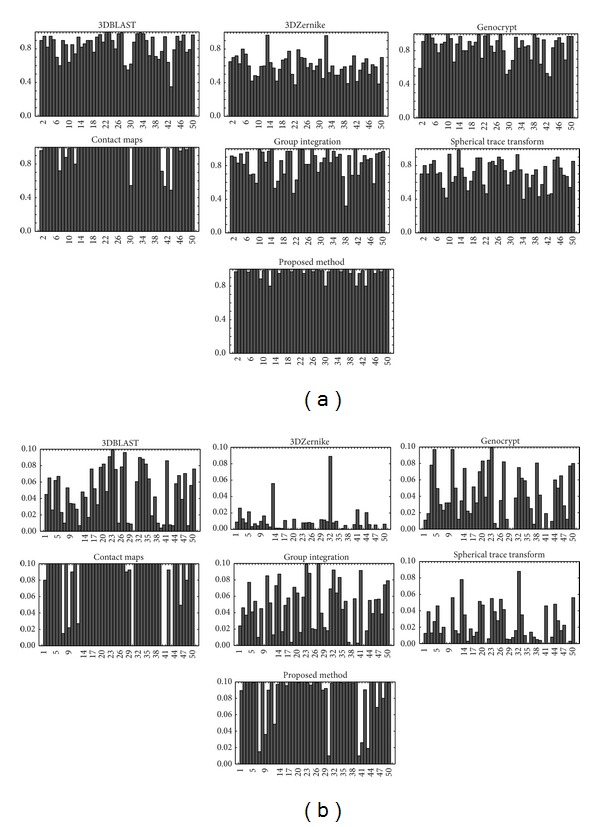
Bar chart for each method. The upper charts show the total AUC, whereas the lower charts show the AUCs calculated for the top 10% of the database. The comparison algorithm is come from literature [12].

Figure 6 shows in a bar graph the query results with each algorithm on 50 protein structures. As Figure 6 shows, the proposed method can easily and successfully identify each query protein, including those that the comparison algorithms failed to identify. Compared with the comparison algorithms, the proposed method may identify some superfamilies more easily. It has a very encouraging and satisfactory result that the comparison algorithms cannot reach. It is worth noting that the classification correctness of the proposed method is almost the same as that that has been done by human experts.

### 3.2. Influence of the Size of the Codebook

In the second experiment, the influence of the vocabulary size (codebook size) upon retrieval performance is studied. The test dataset includes 800 protein structures chosen from SCOP protein database.

**Figure 7:**
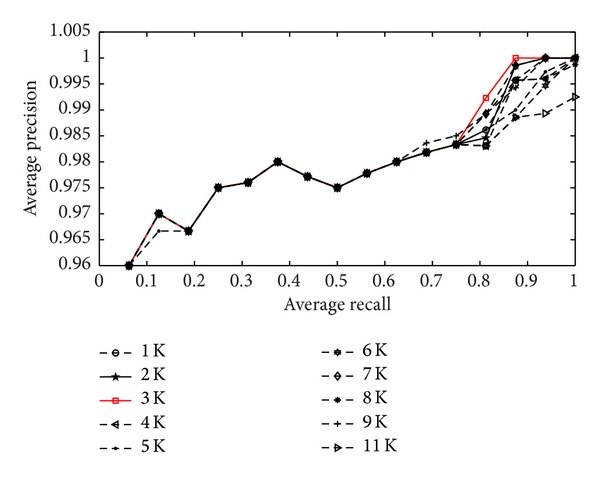
The precision-recall curves of influence of the codebook size.

The number of visual words in the codebook (the codebook size) is a very important parameter in our algorithm. Because the codebook size not only determines the spatial requirement but also significantly affects the retrieval performance.  Figure 7 demonstrates that the precision-recall curves of our methods increase steadily with the codebook size. We observe that with the number of codebook size enlarging from 1,000 to 11,000, the precision-recall values are comparatively stable and the precision rate is between 0.96 and 1 for all codebook sizes. Meanwhile, the difference of precision is 0.02 between the best case and the worst case. So the influence of the number of the codebook on retrieval precision is small. According to the experimental result, we set the number of visual words in the codebook as 3,000 in this paper.

### 3.3. Influence of the Size of the Training Data

**Figure 8:**
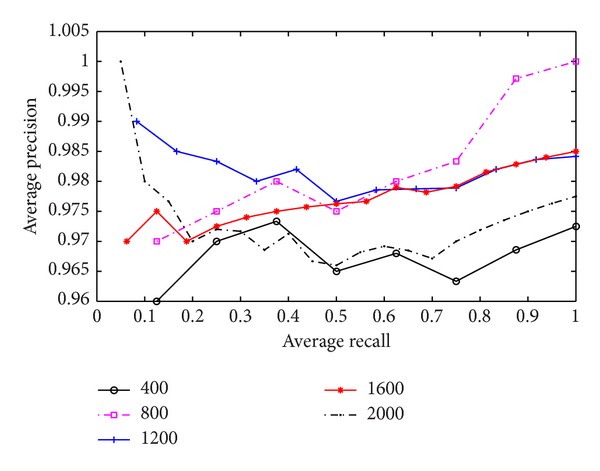
The precision-recall curves of influence of the size of training data.

In the third experiment, we investigate the influence of the training data size on the retrieval efficiency. The test dataset different from the training data is selected from different structural classifications of SCOP protein database.  Figure 8 shows the precision-recall curves when the number of training data is 400, 800, 1,200, 1,600, and 2,000 respectively. As the test results show, the precision rates are all between 0.96 and 1 regardless of the number of training data size.

## 4. Conclusion

In this paper, we proposed a novel feature extracting algorithm for protein retrieval and classification. The proposed method employs a powerful local image feature called SURF and bag-of-visual-feature. The key idea is to describe a view as a word histogram, which is obtained by the vector quantization of the view's local features and to apply KLD to calculate the distance between two models.

A set of experiments were carried out to investigate several critical issues of our method in the CATH and SCOP protein models database from PDB. The experimental results indicate that our method has satisfying performances for protein retrieval and protein categorization that cannot be reached by other comparison methods.

## Figures and Tables

**Table 1:** Nearest neighbour results.

Method	Correct predictions
3DBlast	68%
3DZernike	8%
Genocrypt	56%
Contact maps	80%
Group integration	52%
Spherical trace transform	0%
Proposed method	89%
